# Practical guidelines for radiographers to improve computed radiography image quality

**DOI:** 10.2349/biij.1.2.e12

**Published:** 2005-10-01

**Authors:** N Pongnapang

**Affiliations:** Faculty of Medical Technology, Mahidol University, Bangkok, Thailand

**Keywords:** Computed radiography, image quality, quality control

## Abstract

Computed Radiography (CR) has become a major digital imaging modality in a modern radiological department. CR system changes workflow from the conventional way of using film/screen by employing photostimulable phosphor plate technology. This results in the changing perspectives of technical, artefacts and quality control issues in radiology departments. Guidelines for better image quality in digital medical enterprise include professional guidelines for users and the quality control programme specifically designed to serve the best quality of clinical images. Radiographers who understand technological shift of the CR from conventional method can employ optimization of CR images. Proper anatomic collimation and exposure techniques for each radiographic projection are crucial steps in producing quality digital images. Matching image processing with specific anatomy is also important factor that radiographers should realise. Successful shift from conventional to fully digitised radiology department requires skilful radiographers who utilise the technology and a successful quality control program from teamwork in the department.

## INTRODUCTION

The evolution of medical imaging towards totally digital imaging has accelerated over the past decade [1]. Since its introduction two decades ago, computed radiography (CR) has now become the main player in acquiring, processing and displaying digital images. CR is a process of delivering images that is similar to conventional screen/film system. The main difference between the two systems is that CR processes the optical signals based on a phenomenon called “photostimulated luminescence”, rather than from a prompt emission of light, as in the case with screen-film radiography. In CR, the imaging plate containing storage phosphor is inserted in a cassette similar to a screen-film system, exposed to x-rays, and the signal trapped by the plate read by the scanning of a laser light beam. A photomultiplier tube then enhances the signal coming from the light guide [2,3].

The advantages of CR are its large dynamic range, digital format, portability, and post-processing capability. The technology of CR continues to improve in concomitant with the development of digital technology.

## HOW CR AFFECTS WORKFLOW IN A DIGITAL IMAGING DEPARTMENT

**Figure 1:**
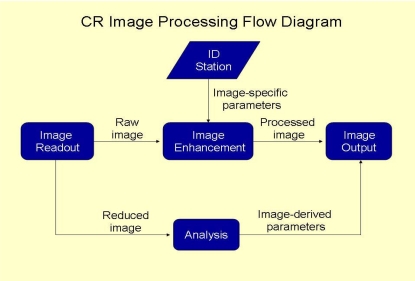
Diagram showing image processing flow in a digital imaging department employing CR technology.

Radiologists and radiographers are the two main professionals involved in the provision of radiology services. An efficient workflow in any radiology department is therefore dependent on how these two professionals plan the department. The present article however, will focus only on the role of the radiographers, which mainly concerns the acquisition of general radiographic projections. This process can be employed using either screen-film or digital radiographic modalities, such as computed radiography. In order to achieve the best of productivity, it is imperative that one has a basic understanding of an efficient workflow. In a digital imaging enterprise, a unique number of tasks make up the process of performing radiographic examinations that could be significantly different from a conventional screen-film system. A common digital imaging workflow includes examination scheduling, patient transportation, patient preparation, data access, examination acquisition, image processing, retrieval of historical comparison studies, and image duplication. The process may also incorporate repeat examinations due to technical factors or loss. Figure 1 shows an image processing work flow in a digital imaging department using CR technology.

## OPTIMISATION OF CR IMAGES

The impression that CR images can always be adjusted after exposing the CR with x-rays is not necessarily true. There are several factors affecting the quality of CR images, and radiographers or technologists are the key persons who are responsible in delivering good quality radiographs, with reasonable radiation dose given to the patients. Quality control of the technical parameters and radiographic positioning are therefore critical to a CR image. Optimisation of a CR image quality may be achieved by optimizing the following factors:

### Positioning and collimation

The routine practice of radiographers includes correct positioning of the organ of interest at the centre and collimating the x-ray field just to cover the organ; this will deliver a good quality image with an acceptable contrast. Proper collimation reduces scattered radiation in the region of interest and reduces the noise that degrades the radiographic contrast. This good practice is still valid with CR, and most image processing software employed in CR relies on the fact that the image collimator edge is detected, so that the contrast may be optimised. Failure of the software to define the image boundary may be caused by a number of factors.

For example, a radiographer may be used to take two projections of a hand radiograph in one 18 cm x 24 cm film. This however, is not a good practice with CR technique, since double or multiple exposures on a single photostimulable phosphor (PSP) can lead to a failure of the image processing software to detect the image boundary. Matching the positioning and collimation with the image processing parameters is also crucial. Some radiographers may take a radiograph of a lumbosacral spine without collimation, thus making the radiograph looks more like an image of the Kidney-Ureter-Bladder (KUB) technique. Image processing will eventually fail to process since the input information is totally different.

### Exposure techniques

In order to introduce CR as a replacement for conventional film-screen technique, the common thinking is that it would be reasonable to adhere to the same exposure techniques to help the radiographers to adapt to the newer technology. But this is not necessarily the case. CR may be operated at a different film speed, and then optimizing the exposure technique accordingly. Existing CR has a speed similar to medium speed film-screen system while spatial resolution is still generally inferior [4].

The idea of reducing radiation dose to patients when switching from screen-film system to CR may not always be valid. To keep the same signal to noise ratio, CR needs 20% more radiation exposure as we treat CR as medium speed film. Reduction of radiation dose to the patient will then results from reduction of reject rate due to poor exposure technique. As a result of poorer intrinsic spatial resolution of the PSP, radiographers need to make sure that when they set up exposure factors, i.e., the mA station from small focal spot should be selected when imaging bones or other high resolution required body parts.

Consideration should be made to the detection efficiency of kVp and K-absorption edge of the PSP, which is totally different from that of the conventional screen-film system. Matching kVp with the pre-set range offered by the image processing is also important. Some radiographers may still use too low kVp for chest radiographs. Employing a standard high kVp technique when the pre-set kVp range for image processing may be higher prevents optimisation of the image quality.

A proper adjustment of exposure technique is therefore still crucial in any radiography practice. Although an increased radiation exposure would yield a higher signal-to-noise ratio and better low contrast detectability in PSP, this would clearly violate the “As low as Reasonably Achievable” (ALARA) principle.

### Image processing selection

**Figure 2:**
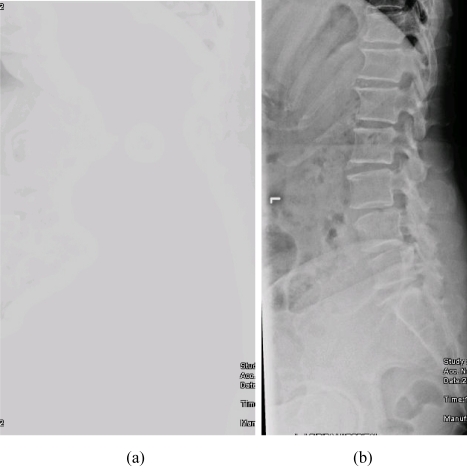
Some examples of artefacts in CR (a) an image with loss of contrast as a result of improper selection of image processing; (b) the same image as 2a shows acceptable image quality as a result of proper selection of image processing.

CR vendors will normally provide various software packages for image processing. Proper selection of an image processing algorithm specific to each type of x-ray examination is thus important. The technical skills of radiographers definitely play a crucial role in determining the quality of the radiographic image. Even though a CR image may be adjusted to improve the image visibility in the cases of over- or under-exposures, it would still be impossible for an image processing to improve the visibility of clinical features that were not available in the raw image. This effect of image processing is illustrated in Figure 2. Image processing may not be substituted for poor positioning techniques and inadequate intrinsic contrast from improper setting of radiographic exposures, or any information outside the edge of the imaging plate for that matter.

### Lifetime of the PSP

One of the major advantages of CR is that the phosphor plate is reusable. However, there are a number of factors that may affect the lifetime of an imaging plate. The plates are subjected to normal wear and tear from scratches, scuffs, cracks, and contamination with dust and dirt, which may interfere with the production of a good image. The establishment of a well-organised quality control program will play an important role in assessing the clinical quality of the imaging plate. This may easily be carried out by artefact assessment and uniformity evaluation across the plate.

## CR ARTEFACTS

The artefacts in radiographic images are seen as any fault impressions visible on the produced radiographic images. These artefacts are distracting and may lead to poor diagnostic accuracy. Although many radiographers may be already accustomed with artefacts appearing in conventional x-ray images, artefacts in CR, require special attention. This is due to the fact that CR artefacts may be produced from various components of the CR system itself [5]. Artefacts may also be generated by the users who are not aware of the proper imaging techniques or selection of appropriate image processing protocols [6,7]. Since CR is also very sensitive to scattered radiation, it is vital that anti-scattered grids be used as in conventional radiography. Radiographers should be concerned of the effects of the aforementioned factors, since these may generate unwanted artefacts that could not be corrected by any image processing algorithm.

**Figure 3:**
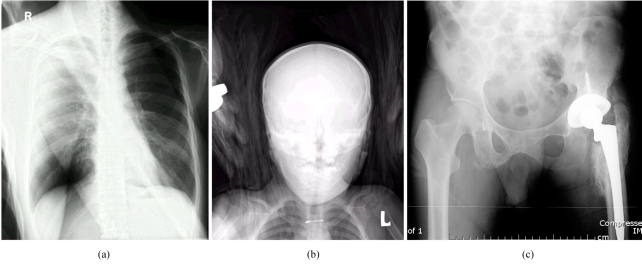
(a) Image artefact resulting from double exposure of the imaging plate. This is a composite image showing a femur superimposed on a chest radiograph; (b) artefact caused by a towel that was used to help in positioning a paediatric patient. Due to the wider dynamic range of CR comparing to conventional film-screen system, radiographic contrast from the towel is readily seen; (c) artefact resulting from dirt collected inside the light-guide in the CR reader leading to the formation of a bright horizontal line (near the bottom of the image).

Implementing a competent quality control program and the proper training of new staff members who will operate the system is therefore still crucial in a digital imaging enterprise. Periodic maintenance from vendors will also contribute to the quality management program by avoiding unwanted circumstances that would degrade the overall quality of the clinical images. Figure 3 demonstrates some common artefacts generated from CR.

## SYSTEM CALIBRATION AND QUALITY CONTROL

To ensure the production of high-quality radiographic images from a CR, a well-organised acceptance testing following the system installation must be carried out. Although the system may have already been calibrated by the manufacturer prior to the installation, the current working environment and conditions in a hospital may be different. Medical physicists will play a role during the acceptance testing by determining that the calibration of the system was made in accordance with the current environment and conditions of the newly-acquired x-ray system. Task Group 18 of the Diagnostic Committee of the American Association of Physicists in Medicine has undertaken the task of establishing a standard of performance for Quality Control (QC) of CR equipment [8].

**Figure 4:**
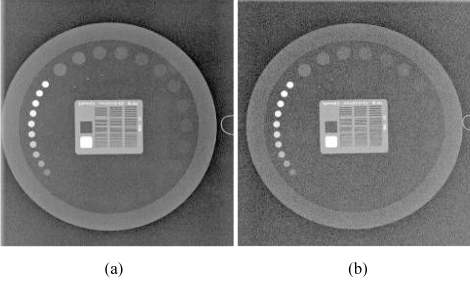
A QC image using a phantom embedded with test patterns such as low and high contrast objects, spatial resolution bar phantom, and gray scale objects (Leeds TOR from the University of Leeds, U.K.). (a) 70 kVp, 2 mAs; (b) 70 kVp, 0.5 mAs. Note the increase in noise level.

A periodic quality control program is still necessary even after a successful completion of an acceptance testing. The medical physicist is responsible for performing acceptance testing and setting up the quality control program for the CR system. QC processes for CR are no less important than they are for conventional screen-film radiography. The design of the program needs to be modified to fit the differences that are unique to the characteristics of the CR and good quality control program needs cooperation between radiographers and medical physicists. Radiographers perform daily and periodic check of quality control items that do not require complicated dose measurement procedures or reject analysis and image quality evaluation. Medical physicists should be responsible for performing the review of QC activities, patient dose assessment and annual quality assessment of the CR system. Figure 4 shows an example of images obtained from an image quality phantom (Leeds TOR, University of Leeds, U.K.). Table 1 summarises a QC program for CR listing the various tasks, frequency and individuals to be responsible.

**Table 1:** Quality control program for a computed radiography system

Frequency	Tasks	Responsibility
Daily	System inspection for physical defects	Radiographer
	Physical inspection of display devices	
	Secondary erasure of imaging plates	
	Verification of system interface/network	
Weekly	Verification of displayed images	Radiographer
	Phantom image quality control testing	
	- Image quality	
	- Artefacts	
Monthly	Inspect and clean image receptors	Radiographer
	Review image rejection rate	
	QC review for ‘out-of-tolerance’ issues	
Semi-annually/Annually	Evaluate image quality and patient dose	Medical physicist
	Acceptance tests to re-establish baseline value	
	Review for:	
	- Patient exposure trends	
	- Retake activity	
	- QC records	
	- Service history	

## CONCLUSION

Practical guidelines for better image quality in computed radiography is mainly concerned with the professional skills of the users and the establishment of an efficient quality control program specifically designed to produce the best quality of clinical images. Another important factor is the level of teamwork among the users. Radiologists should support and encourage staff in the radiology department to appreciate the importance of an effective quality control program. In addition, radiographers who utilise the technology should also receive proper training on developing professional skills concerning CR technology and must also play an important role in the quality control program. A successful digital radiology enterprise will undoubtedly earn immeasurable benefits from an effective quality control program and skilful radiographers who correctly utilise the technology.
